# Emergence Agitation and Anesthetic Considerations in the Management of Patients With Post-Traumatic Stress Disorder: A Report of Two Cases and a Review of the Literature

**DOI:** 10.7759/cureus.33794

**Published:** 2023-01-15

**Authors:** Jordan Huang, Nitin Chopra, Natesh Yepuri, Sudhakar Kinthala

**Affiliations:** 1 Anesthesiology, Guthrie Robert Packer Hospital, Sayre, USA

**Keywords:** dexmedetomidine, emergence delirium, emergence agitation, post-traumatic stress disorder, anesthetic management

## Abstract

Post-traumatic stress disorder (PTSD) is a psychological disturbance resulting from exposure to a traumatic experience that lasts more than one month. PTSD in the United States has a lifetime prevalence of 3.4% to 26.9% in civilians and 7.7% to 17.0% in military veterans. Emergence agitation (EA) and emergence delirium (ED) are known phenomena in the postanesthetic period. PTSD is closely associated with EA following anesthesia. In addition, EA in patients with PTSD can be severe and challenging to manage. EA is a risk to both patients and healthcare workers.

Furthermore, EA has been shown to increase the overall risk of postoperative delirium and complications. Currently, studies on the anesthetic management of patients with PTSD are scarce and limited to case reports. Here, we present a summary of several important published case reports and a brief review of the literature regarding the anesthetic management of PTSD and EA to aid in managing patients with PTSD. In addition, we present two cases of successful EA prevention in patients with severe PTSD. From our review of the literature and the successful prevention of EA in our patients with severe PTSD, we conclude that there is an increased need for overall awareness among anesthesia and perioperative care providers of the effect of PTSD on EA. Anesthesia providers should aim to include as many management recommendations as possible and avoid possible triggers of EA via a multidisciplinary approach. Multiple pharmacological agents have been used for the anesthetic management of PTSD with varying results. Of the agents studied, dexmedetomidine has been found to be the most consistently beneficial.

## Introduction

Post-traumatic stress disorder (PTSD) is defined as a psychological disturbance of greater than one month, occurring after exposure to a traumatic experience (the Diagnostic and Statistical Manual of Mental Disorders, Fifth Edition, or DSM-V, criteria) [1]. It is believed that the lifetime prevalence of PTSD in the United States is 3.4% to 26.9% in civilians and 7.7% to 17.0% in military veterans [2].

Emergence agitation (EA) and emergence delirium (ED) are known phenomena that occur in the postanesthetic period. PTSD is closely associated with EA following anesthesia; moreover, EA in patients with PTSD can be severe and challenging to manage [3-7]. EA is believed to occur in 3.0% to 21.3% of adults after general anesthesia [8].

EA and ED are both defined terms in the literature, and both terms are often used interchangeably. Wilson defined ED as a situation in which the patient "is emerging from general anesthesia and is subsequently seen thrashing around violently...screaming, speaking incoherently, hitting, biting, or attempting to leave the operating room...encompassing any time frame from the end of surgery to discharge from the PACU” [9]. EA is defined as a “brief, self-limited nonfluctuating state of psychomotor excitement immediately surrounding emergence from general anesthesia” [3]. The distinguishing feature of EA from ED is that, in EA, the patient must be transitioning into a state of consciousness during the agitation episode rather than becoming agitated after a sense of consciousness has already been established [3].

EA is a risk factor for both patients and healthcare workers. It can cause extremity injuries, surgical site disruption, hemorrhage, unintended extubation, and the traumatic removal of venous, arterial, epidural, and urinary catheters. Furthermore, EA has been shown to increase the overall risk of postoperative delirium, complications, length of operating room time, and length of stay in the postanesthesia care unit (PACU) [3,4,9]. Currently, studies on the anesthetic management of patients with PTSD are scarce and limited to case reports. Here, we present a summary of all published case reports and a brief review of the literature regarding the anesthetic management of PTSD and EA to aid in the management of this phenomenon in patients with PTSD. In addition, we present two cases of successful EA prevention in patients with severe PTSD.

## Case presentation

Written permission was obtained from the patients for the publication of the case reports.

Case 1

A 37-year-old man with a past medical history (PMH) of severe PTSD from prior military deployments, who was on amitriptyline for severe PTSD symptoms and classified as American Society of Anesthesia (ASA) physical status 2, was scheduled for robotic cholecystectomy under general anesthesia. Given the high risk of EA due to severe PTSD, he was treated with dexmedetomidine prophylactically preoperatively and intraoperatively for EA. The patient also received preoperative midazolam for anxiolysis. Under standard ASA monitoring, general anesthesia was induced with propofol and maintained with sevoflurane. To avoid arousal, the operating and recovery rooms were maintained in a quiet, nontriggering environment. The patient had an uneventful emergence from anesthesia and the postoperative course.

Case 2

A 52-year-old woman with a PMH of hypothyroidism, severe PTSD from a prior physical assault, and classified as ASA physical status 2 was scheduled for elective shoulder arthroscopy, subacromial decompression, and capsular release. The patient reported experiencing daily night terrors and flashbacks. Her medications included hydroxyzine, gabapentin, sumatriptan, amitriptyline, and meclizine. Due to her history of severe PTSD, a multidisciplinary approach involving a nonstimulating environment and trigger avoidance for EA was planned. A total of 20 mcg of dexmedetomidine was given in 10 mcg increments preoperatively. The patient underwent a preoperative interscalene brachial plexus block for intraoperative and postoperative analgesia. With standard ASA monitoring, general anesthesia was induced with propofol and maintained under total intravenous anesthesia (TIVA) with propofol infusion. For EA prophylaxis, 10 mcg of dexmedetomidine and 20 mg of ketamine were administered 10 minutes before planned emergence. She had an uneventful emergence from anesthesia and recovery without any EA flashbacks. However, at the 24-hour postoperative follow-up, she reported a single PTSD flashback the previous evening, along with the return of her baseline terrors.

## Discussion

PTSD is characterized by a re-experiencing of traumatic events with persistent arousal, hypervigilance, avoidance, emotional numbing, and difficulty with cognition, leading to significant functional impairment. In the civilian population, the PTSD-inciting trigger is typically a single-incident blunt or penetrating trauma, assault, fall, gunshot wound or stabbing, or sexual abuse [1]. Patients with a prior history of alcohol and substance misuse, psychiatric problems, anesthetic awareness, or prolonged hospitalization in intensive care units have also been found to be at high risk of developing PTSD [1,2,10].

The proposed pathophysiological alteration in PTSD is glutaminergic hyperactivity due to impaired regulation of principal neurons in the basolateral amygdala by local GABAergic neurons [3]. The mechanism of EA is not well understood. A neuroinflammatory mechanism is thought to be a contributory factor, as the incidence of EA has been found to be higher in patients with higher levels of inflammatory markers and endogenous catecholamines. Furthermore, increased glutamate neurotransmission is associated with stressful states. An *amygdalocentric* model for the pathophysiology of EA has been proposed. Therefore, it is possible that glutaminergic modulation may be a viable mechanism for the treatment of both PTSD and EA.

Alterations in the amygdalocentric neurocircuitry (AN) of the brain under general anesthesia and benzodiazepines have been proposed as the underlying mechanism for EA in PTSD patients, where the *free flow* of fear is transmitted through the AN to the brainstem and hypothalamic regions of the brain [4]. Additionally, alterations in the hippocampus, medial prefrontal cortex, and amygdala increase the propensity for hyperarousal and agitation [10].

Anesthetic management and prevention of EA in patients with PTSD have not been extensively studied, and no standardized protocols currently exist. A thorough review of the literature (we searched the MEDLINE, Scopus, and Google Scholar databases for articles on the anesthetic management of PTSD) found only 12 publications, eight of which are case reports (a total of 15 cases), four expert opinion articles, and no prospective studies. In the case reports, anesthetic management strategies varied significantly (Table 1).

**Table 1:** Summary of case reports of anesthetic management of PTSD patients. *Case with the corresponding number listed only for case reports depicting more than one anesthetic case. **Unknown, i.e., authors did not specifically discuss the postoperative management in the case report. ***No PTSD history was disclosed on the initial patient evaluation, but the patient was later found to be on amitriptyline and sertraline, which are concerning medications for the treatment of PTSD. ****No PTSD history was disclosed on the initial patient evaluation, but the patient was found to have symptoms significant for PTSD after recovering from an agitation episode. M, male; F, female; YO, years old; EA, emergence agitation; ED, emergence delirium; PTSD, post-traumatic stress disorder; MAC, monitored anesthesia care; PACU, post-anesthesia care unit; TIVA, total intravenous anesthesia

Author (Year) Case* [Reference number]	Age/sex	Surgery	Type of anesthesia	Psychiatric illnesses, risk factors for EA, and psychiatric medications	Anesthetic history	Preoperative management	Intraoperative management	Postoperative management	Outcome, course, and use of rescue agents
Mashour et al. (2006) [11]	Adult/F	Colporrhaphy, suburethral sling, cystoscopy, and gynecologic examination under anesthesia	Spinal with MAC	PTSD and no medications disclosed	Awareness under anesthesia	Midazolam	Intrathecal hyperbaric bupivacaine/fentanyl and grounding technique	Unknown**	No EA or ED or no flashbacks or pain during recovery
Crosby et al. (2007) [12]	Adult/F	Unknown	General	PTSD, depression, and no medications disclosed	Unknown	Midazolam	Fentanyl and propofol	Midazolam	EA and flashbacks presented and treated with propofol; patient given private room and female nurse; the patient provided with own primary care physician, grounding techniques, reorientation, and assurance
Lovestrand et al. (2013) Case 1 [13]	30 YO/M	Right inguinal hernia repair	General	PTSD, on amitriptyline and sertraline***	Unknown	None	Midazolam, fentanyl, lidocaine, propofol, and hydromorphone	Unknown**	EA presented with flashbacks, attempted reorientation with military staff and wife, and reoriented after receiving ondansetron and promethazine for post-operative nausea/vomiting
Lovestrand et al. (2013) Case 2 [13]	21 YO/M	Bilateral breast reduction	General	PTSD, traumatic brain injury, and no medications disclosed	Unknown	Minimizing noxious stimuli and maintaining nonstimulating environment during induction/emergence, adequate analgesia, and same nurse/anesthetist involved for the whole case	Midazolam, fentanyl, propofol, clonidine, localization of wound with bupivacaine by surgeon, sevoflurane	Deep extubation, minimally stimulating environment in PACU, military personnel available for reorientation, analgesics available, and family and friends allowed at the bedside when appropriate	No EA or ED, smooth emergence and postoperative course
Shoum (2014) Case 1 [14]	Adult/M	Esophagogastroduodenoscopy	MAC	PTSD**** and no medications disclosed	EA with prior sedation	Midazolam	Propofol	Attempted gentle, unstimulated awakening	EA present with self-removal of monitors and intravenous line as well as leaving hospital premises; the patient reoriented by his wife’s best friend and accompanied to PACU and discovered to have intense nightmares since being stabbed at 16 YO
Shoum (2014) Case 2 [14]	67 YO/M	Urgent total hip replacement	Spinal with MAC	PTSD, chronic pain, depression, alcohol abuse, frequent nightmares; on fentanyl patch, lorazepam, and butalbital/aspirin/caffeine	EA with fighting and punching	Ketamine	Intrathecal hyperbaric bupivacaine/epinephrine	Unknown**	No EA; alert and oriented in PACU
Nguyen et al. (2016) Case 1 [5]	58 YO/M	Open reduction internal fixation of distal radius fracture	General	PTSD, anxiety, depression, polysubstance dependency, and no medications disclosed	Wild wake-ups, agitation, aggression, and emotional liability on emergence	Unknown	Fentanyl, propofol, hydromorphone, and sevoflurane	None	Severe ED present; treated with propofol, was less agitated upon hearing spouse’s voice
Nguyen et al. (2016) Case 2 [5]	63 YO/M	Carpal tunnel release and excision of hand lipoma	General	PTSD, bipolar disorder, anxiety, depression, chronic back pain, and no medications disclosed	Violent wake-ups	Dexmedetomidine	Propofol, fentanyl, dexmedetomidine, sevoflurane, and wound infiltration with bupivacaine	Verbal coaching and controlled noise levels	No ED
Gentili et al. (2017) Case 1 [10]	62 YO/M	Disc hernia	General	PTSD and no medications disclosed	Unknown	Unknown	Propofol, remifentanil, and sevoflurane	Unknown**	ED present, treated with midazolam and no recollection of an ED episode
Gentili et al. (2017) Case 2 [10]	65 YO/M	Colonoscopy	General	PTSD and no medications disclosed	Prolonged paralysis from prolonged curarization during prior anesthesia	Unknown	Propofol, remifentanil, and sevoflurane	Unknown**	ED present, treated with midazolam and no recollection of an ED episode
Gentili et al. (2017) Case 3 [10]	58 YO/M	Colonoscopy	General	PTSD and no medications disclosed	Unknown	Unknown	Propofol, remifentanil, and sevoflurane	Unknown**	ED present, treated with rescue midazolam and no recollection of an ED episode
Read et al. (2017) Case 1 [15]	33 YO/M	Two-level anterior cervical discectomy and fusion	General	PTSD and on gabapentin and amitriptyline	No previous complications	Midazolam	TIVA with propofol, remifentanil, ketamine, and hydromorphone	Unknown**	ED present, verbal coaching; bringing a spouse to PACU unsuccessful, rescue dexmedetomidine successful
Read et al. (2017) Case 2 [15]	40 YO/F	Open reduction internal fixation of ankle fracture	General	PTSD, paranoid schizophrenia, substance abuse (cocaine, alcohol, tobacco); on quetiapine, amitriptyline, lorazepam, methadone, and oxycodone	No previous complications	Midazolam	Propofol, hydromorphone, and sevoflurane	Unknown**	ED present, fentanyl and hydromorphone unsuccessful, and rescue dexmedetomidine successful
Read et al. (2017) Case 3 [15]	49 YO/M	Single-level posterior lumbar interbody fusion	General	PTSD, insomnia, migraines, chronic back pain, on escitalopram	No previous complications	Midazolam	Propofol, fentanyl, hydromorphone, and sevoflurane	Unknown**	ED present; midazolam, fentanyl, and valium unsuccessful; rescue dexmedetomidine successful
Marenco-Hillembrand et al. (2020) [16]	34 YO/M	Awake craniotomy	General-MAC-general	PTSD, anxiety, depression, on sertraline	Unknown	Unknown	Bilateral scalp blocks, propofol, fentanyl, and dexmedetomidine infusion for the awake phase; excessive stimuli and extraneous noises minimized; and the neuropsychologist reoriented the patient upon waking	Unknown**	No PTSD flashbacks postoperatively

The mixed results of many treatment strategies have demonstrated the multifactorial nature of EA. However, it does appear that dexmedetomidine is consistently helpful in addressing the multifactorial nature of PTSD, as it provides both sedation and analgesia and smooths emergence [3,5,11]. Although expert opinion suggests avoiding benzodiazepines in patients with PTSD, midazolam has been used by most providers in these case reports with varying effects [3,4,6,11-15].

Upon reviewing all published reports, it is evident that the management of EA is heterogeneous. In most cases, many areas of care could have included components of recommendations that could potentially help in the prevention of EA but did not include them. This shows a limited understanding of PTSD and EA among anesthesia practitioners.

Similarly, expert opinion articles on the management of EA in patients with PTSD are limited, with all four articles studying only the military population. However, these articles agree with the proper identification of patients at risk of EA, minimization of anxiety about the upcoming procedure and flashbacks, maintenance of a minimally stimulating environment, and intraoperative sympatholytic therapy [3,4,6,7].

Pharmacological agents used in the management of PTSD include selective serotonin reuptake inhibitors, tricyclic antidepressants, prazosin, nefazodone, and mirtazapine. A review of common medications used for PTSD is important, as there can be potential for medication interactions, QT interval prolongation, and increased drug metabolism through liver enzyme induction [1]. Tricyclic antidepressants, for example, can further exacerbate the inherent QT interval prolonging effects of commonly used anesthetic agents such as propofol and inhalational anesthetics, as well as antiemetics like ondansetron and metoclopramide. Also notable is mirtazapine, which has alpha-2 receptor antagonist activity and can, therefore, interfere with the effects of dexmedetomidine.

It is essential to develop rapport with at-risk patients once they are identified. It is also important to inquire about the patient’s anesthetic history, with any prior episodes of *wild wake-ups*, alerting the provider to patients at elevated risk of experiencing EA. Including a patient's family member in the process of explaining and developing the anesthetic plan may be helpful. Furthermore, identifying any concerns and sources of anxiety for the patient, helpful coping mechanisms for the patient during stressful times, and avoiding any potential flashback triggers can assist in creating a perioperative environment that is as nonstimulating for the patient as possible. Having family members or trusted friends present in the perioperative environment is extremely helpful in providing reassurance to the patient as well as orienting patients to their present surrounding environment. Although benzodiazepines do reduce anxiety, most expert opinion articles agree that benzodiazepines preferably be avoided [3,4,6].

Regarding intraoperative management, expert opinion generally agrees that minimizing external stimuli throughout the perioperative phase is essential to prevent flashbacks. It is important to alert the operating room staff to the patient's existing PTSD and maintain a quiet environment in the operating room during induction and emergence. As unfamiliar environments have been identified as potential triggers of PTSD flashbacks, using the same anesthesia and nursing providers throughout the patient's perioperative course are also suggested to give the patient a sense of familiarity and comfort. Furthermore, additional safety measures such as securing all indwelling catheters, airway devices, and wound dressings and having a low threshold for calling another anesthesia provider and also support staff for assistance in the event of severe agitation may be helpful [3,4,6].

The choice of a specific anesthetic technique may not be generalizable to all patients with PTSD, and individual patient factors should be considered. It is also unclear whether total intravenous anesthesia rather than inhalational anesthesia is clinically effective in preventing EA in patients with PTSD [3,4,6,8,15]. It is also debatable whether avoiding general anesthesia when feasible can lower the risk of EA in patients with PTSD. The successful use of regional anesthesia-based techniques to prevent EA in patients with PTSD has also been documented in the literature [11,14]. Irrespective of the anesthetic technique implemented, the consensus is that providing sedation and adequate analgesia throughout the perioperative period is essential.

The current expert opinion suggests that dexmedetomidine and ketamine are likely to be effective pharmacological agents in the management of patients with PTSD in the perioperative period, with evidence for dexmedetomidine's efficacy being particularly strong in the literature. Dexmedetomidine and clonidine are alpha-2 adrenergic agonists that reduce sympathetic outflow, thereby providing sedation and some analgesic properties, as well as slowing emergence, thus preventing the triggering of a flashback. The utility of dexmedetomidine throughout the perioperative period is supported by studies demonstrating its effectiveness in reducing EA in patients undergoing nasal surgery, as well as a case report demonstrating its effectiveness as a rescue medication for patients experiencing EA [15]. A recent prospective study also suggested that dexmedetomidine effectively decreased pediatric anesthesia emergence delirium (PAED) scores in military veterans with high preoperative anxiety [17]. However, recent literature on the use of dexmedetomidine in cardiac surgery patients suggests that it may increase the risk of delirium in this patient population [18].

Ketamine, on the contrary, is a dissociative anesthetic that acts as an antagonist of N-methyl-D-aspartic acid (NMDA) receptors, which open when bound by glutamate. Given the likely glutaminergic mechanism of PTSD and EA, one may thus extrapolate that a subanesthetic dose of ketamine (0.15-0.5 mg/kg) may decrease EA in patients with PTSD [7]. Ketamine is gaining acceptance as a treatment for patients with severe PTSD. Furthermore, some studies have demonstrated the effectiveness of ketamine in reducing EA in patients undergoing nasal surgery [19]. In contrast, a multicenter randomized clinical trial investigating the use of ketamine in preventing emergence delirium in 672 adults with a mean age of 70 years found that patients receiving ketamine doses of 0.5 and 1.0 mg/kg had no difference in delirium incidence compared to controls. The study found that patients receiving ketamine had a higher incidence of postoperative hallucinations, suggesting that routine use of subanesthetic doses of ketamine may be harmful [20].

If an EA episode occurs, the current management of EA includes reassurance and reorientation. It is important to rule out potential alternative causes of agitation such as hypoxemia, hypercarbia, hypoglycemia, electrolyte imbalances, hypothermia, seizures, strokes, and alcohol or opioid withdrawal. Serotonin syndrome should also be considered, given that many patients with PTSD are on serotonergic medications. After reversible causes are treated, repeat dexmedetomidine loading may also be helpful [3,4,6,16]. Low-dose propofol boluses may be used to *smooth* reemergence after a failed initial attempt [3,4,5,6,12]. Small doses of haloperidol may also be administered [3,4,9]. It is also essential to consider the residual effects or toxicity of anesthetic drugs, inadequately controlled pain, and bladder distention as possible causes. *Grounding techniques* are also recommended by Veterans Association psychologists to not only reorient patients experiencing flashbacks but also give patients a sense of control [1]. Most expert opinion articles tend to suggest avoiding midazolam because of its potential for causing dissociation and worsening agitation episodes despite a lack of evidence [3,4,6]. Furthermore, it is believed that the decreased number of binding sites on gamma-aminobutyric acid (GABA) receptors in PTSD patients may limit the effectiveness of benzodiazepines in this patient population [7].

Once an EA episode has subsided, the patient is asked to verbally share what they experienced during the episode. Accurate documentation of the EA event and surrounding circumstances is also essential, as it provides a framework for preventing further episodes in subsequent anesthetic environments. Follow-up phone calls after discharge may also help identify additional areas of improvement and possible referral to behavioral health services [3,4,6].

Both our cases were successful in that EA and perioperative flashback events were successfully prevented through a quiet environment during induction, emergence, and the postoperative setting, as well as through the administration of dexmedetomidine, in line with current recommendations. However, the anesthetic management in each case had some key differences (Table 2). Notably, the anesthetic plan in the first case was devised before our literature review, based on the clinical experience of the anesthesiologists taking care of the patient. This is reflected in the fact that both an inhalational anesthetic and midazolam were used, despite both not being considered ideal agents in current recommendations.

**Table 2:** Summary of anesthetic management of PTSD patients from this report. M, male; F, female; YO, years old; EA, emergence agitation; PTSD, post-traumatic stress disorder; TIVA, total intravenous anesthesia

Case number	Age/Sex	Surgery	Type of anesthesia	Psychiatric illnesses, risk factors for EA, and psychiatric medications	Anesthetic history	Preoperative management	Intraoperative management	Postoperative management	Outcome, course, and use of rescue agents
Case 1	37 YO/M	Robotic cholecystectomy	General	PTSD, anxiety, on amitriptyline and promethazine	None prior	Midazolam, dexmedetomidine, controlled noise levels during induction	Dexmedetomidine, sevoflurane	Controlled noise levels during the emergence	No EA, no PTSD flashbacks postoperatively
Case 2	52 YO/F	Arthroscopic capsular release and subacromial decompression with acromioplasty	Preoperative interscalene block and general	PTSD, anxiety, night terrors; on hydroxyzine, gabapentin, sumatriptan, amitriptyline, meclizine, and lidocaine transdermal patch	No known history of EA with prior general anesthesia	Dexmedetomidine, propofol, interscalene, and superficial cervical plexus blocks with 0.5% ropivacaine, controlled noise levels during induction	Dexmedetomidine, ketamine, propofol TIVA	Controlled noise levels during the emergence	No EA, no PTSD flashbacks immediately postoperatively, one episode of PTSD flashbacks in the evening on the day of surgery post-discharge consistent with the patient’s baseline

In the second case, we aimed to implement as many components of the current recommendations and avoid as many triggers of EA as possible via a multidisciplinary approach. We avoided midazolam and administered dexmedetomidine for preoperative sedation and anxiolysis while performing the interscalene block. TIVA, ketamine, and dexmedetomidine for emergence were administered to successfully prevent EA. The fact that EA was successfully prevented in both cases reflects the multifactorial nature of EA in patients with PTSD.

Owing to the poorly understood nature of the mechanism behind EA and the limited evidence available in its management, the relevant recommendations are yet to be validated. It is essential to investigate the role of individual components in the management of EA in patients with PTSD to draw concrete conclusions regarding the appropriate management of this condition.

## Conclusions

Our conclusions are as follows: (1) there is a need for increasing overall awareness of the effect that PTSD has on EA among anesthesia and perioperative care providers; (2) the anesthesia provider should aim to implement as many management recommendations in the expert opinion articles as possible, with particular focus on providing adequate perioperative sedation and analgesia and identifying and avoiding all possible triggers of flashbacks via a multidisciplinary approach; (3) dexmedetomidine addresses the multifactorial nature of PTSD and appears to be helpful in multiple care phases; and (4) the roles of midazolam and ketamine in the management of patients with PTSD are unclear, although some practitioners have successfully used these drugs in the anesthetic management of patients with PTSD.
